# Effectiveness of educational interventions for improving healthcare professionals' information literacy: A systematic review

**DOI:** 10.1111/hir.12562

**Published:** 2025-02-02

**Authors:** Mauricette Moling Lee, Xiaowen Lin, Eng Sing Lee, Helen Elizabeth Smith, Lorainne Tudor Car

**Affiliations:** ^1^ Lee Kong Chian School of Medicine Nanyang Technological University, Singapore and Health and Social Sciences, Singapore Institute of Technology Singapore Singapore; ^2^ Lee Kong Chian School of Medicine Nanyang Technological University Singapore Singapore; ^3^ Family Medicine and Primary Care, Lee Kong Chian School of Medicine Nanyang Technological University, Singapore and National Healthcare Group Polyclinics Singapore Singapore; ^4^ Family Medicine and Primary Care, Lee Kong Chian School of Medicine Nanyang Technological University Singapore Singapore; ^5^ Lee Kong Chian School of Medicine Nanyang Technological University, Singapore and Imperial College London London UK

**Keywords:** allied health, clinical questions, doctors, education and training, evidence‐based medicine (EBM), evidence‐based nursing (EBN), evidence‐based practice (EBP), health professionals, information literacy, nurses

## Abstract

**Background:**

It is unclear which educational interventions effectively improve healthcare professionals' information literacy.

**Objectives:**

We aimed to evaluate the effectiveness of educational interventions for improving the formulation of answerable clinical questions and the search skills of healthcare professionals.

**Methods:**

We followed the Cochrane methodology and reported according to the PRISMA statement. The following databases from inception to November 2022: MEDLINE, Cochrane CENTRAL, EMBASE, Web of Science, CINAHL, and Google Scholar search engine, were searched. Randomised controlled trials and crossover trials on any educational interventions were included. Studies on search tools that are obsolete were excluded.

**Results:**

Ten studies that mainly compared the effectiveness of lectures and bedside education to lectures or no intervention for searching of PubMed and/or MEDLINE, were included. There was evidence for improved attitude towards the intervention favouring lecture with self‐directed learning over lecture, bedside education, and computer‐assisted self‐directed learning (RR: 1.14; 95% CI 1.06–1.23; *N* = 2 studies; 1064 participants; *I* ^2^ = 0%; moderate certainty evidence). There were limited findings on the knowledge, skills, satisfaction, and behaviour outcomes.

**Conclusion:**

Future research should include a wider set of outcomes, be reported better and explore the use of digital technology for delivery of educational interventions. Further research should entail well‐designed trials with relevant outcomes evaluating novel digital‐based educational interventions.

**Key Messages**

- Health libraries and information professionals should incorporate pre‐appraised evidence searching and use training for clinicians in their information literacy training. Librarians should assess the effectiveness of information literacy training they provide to healthcare professionals due to the limited and unclear evidence in this area.
- Future studies on information literacy educational programmes should investigate the use of digital technologies in the delivery of information literacy training.
- Future studies should aim to employ validated measurement instruments to assess outcomes of information literacy training to ensure the reliability and comparability of the findings.

## INTRODUCTION

Evidence‐based medicine (EBM) is ‘the conscientious, explicit, and judicious use of current best evidence in making decisions about the care of individual patients’ (Sackett et al., 1996). It consists of five steps: (1) formulating answerable clinical questions; (2) finding the evidence; (3) appraising the evidence; (4) applying the evidence; and (5) evaluating performance (Sackett, 1997). Research has shown that evidence‐based information can improve patient outcomes (Fraser & Poole, 2022; Stephens et al., 2016) and inform clinicians' decision‐making process in clinical practice. Clinicians frequently generate questions during patient encounters, but these questions do not always compel them to search the literature (Del Fiol et al., 2014). Many questions remain unanswered due to diverse challenges, including a lack of skills in developing questions, crafting effective search strategies, and accessing databases to identify the best available scientific evidence (Del Fiol et al., 2014). This can negatively affect the quality of care provided by clinicians (Bruin‐Huisman et al., 2017; Cahir et al., 2014).

Information literacy includes the identification of information needs, the creation of searchable clinical questions, the performance of effective search techniques, and the retrieval of scientific evidence (Ross, 2010). These skills are essential for the implementation of EBM (Azami et al., 2020; Janavi et al., 2018; Mokhtar et al., 2012). Forming clinical questions and addressing them is an integral part of clinical decision‐making and a prerequisite for clinicians to advance patient care (American Library Association, 2013; Amiel et al., 2021; College of Family Physicians Singapore, 2022; Daei et al., 2020). A good clinical question must address the problem precisely with the best scientific evidence available (Carneiro, 1998). A useful framework for developing answerable clinical questions is ‘PICO’, an acronym for Population, Intervention, Comparison(s), and Outcome (Higgins et al., 2022). In addition to improving how they formulate their clinical questions, clinicians also need to be competent in the search for and identification of relevant evidence in the literature (Barzkar et al., 2018; Oliveri et al., 2004). Identifying the best available scientific literature to answer clinical questions is accomplished by searching electronic bibliographic databases such as MEDLINE, EMBASE, the Cochrane Library, and more. However, several barriers prevent healthcare professionals from seeking the best available scientific evidence (Brassil et al., 2017; Ely et al., 2002; Green & Ruff, 2005; Sadeghi‐Bazargani et al., 2014). Among the barriers are a lack of confidence in formulating good clinical questions or developing an appropriate search strategy to identify relevant medical literature, as well as unfamiliarity with bibliographic databases (Brassil et al., 2017; Ely et al., 2002; Green & Ruff, 2005; Sadeghi‐Bazargani et al., 2014). Since clinical questions should be answered with the best available scientific evidence, it is also important for clinicians to be familiar with different levels of evidence and the suitability of different study designs to answer the clinical questions (Petrisor & Bhandari, 2007). Such knowledge can be used to include study design filters in a search strategy, improving the effectiveness of literature searches (Wallace et al., 2022). The use of digital technology among healthcare professionals worldwide is becoming more common and is an increasingly important channel for retrieving clinical information (Johnson et al., 2016). Smartphones, in particular, enable easy connectivity with peers and instant access to numerous electronic resources and a vast amount of information (Gagnon et al., 2010). Smartphones, which allow access to internet websites and apps such as UpToDate (Wolters Kluwer, 2023), can therefore be used to support evidence‐based practice and information‐seeking in many ways (Kwag et al., 2016; Lee Ventola, 2014). In addition, pre‐appraised sources of evidence such as UpToDate, with clear recommendations on the quality of evidence and its applications in clinical practice, are becoming increasingly common and are helpful resources for busy clinicians. While the third and fourth step of EBM might no longer be as important with the use of pre‐appraised sources of evidence, information literacy, that is, ability to formulate a clinical question and develop a relevant search strategy are still essential.

The development of EBM skills, including information literacy, in medical students is well‐studied (Maggio et al., 2013; Romero‐Robles et al., 2022). There are also systematic reviews on the effectiveness of EBM training for healthcare professionals and nurses (Hecht et al., 2016; Sapri et al., 2022). However, these reviews focused on all five steps of the EBM model, which encompassed other information literacy skills, such as a critical appraisal of different study designs and interpretation of the study findings. These reviews included studies on educational interventions which spanned several EBM steps and provided an estimate of the effects of training on the overall EBM‐related outcomes such as knowledge, skills, and attitudes. The aim of this review is to complement existing literature by assessing the effectiveness of educational interventions for improving information literacy exclusively in healthcare professionals. More specifically, this is a systematic review of educational interventions to improve healthcare professionals' ability to carry out the first two steps of EBM, namely, formulate answerable clinical questions and find evidence. The objective of this systematic review was to answer the following research questions: What are the effective educational interventions for improving healthcare professionals' formulation of answerable clinical questions and searching for evidence?

## METHODS

This systematic review was conducted using the Cochrane methodology and reported according to the Preferred Reporting Items for Systematic reviews and Meta‐Analyses statement (PRISMA) (Higgins et al., 2022; Page et al., 2021). The protocol for this systematic review was registered on PROSPERO [CRD 42022381597] on 16 December 2022 (Lee et al., 2022).

### Criteria to select studies to be included in the review

Given the methodological rigour of randomised controlled trials (RCTs) and to minimise bias, our systematic review included RCTs and crossover trials involving any educational interventions of any duration or frequency on information literacy in healthcare professionals. Data were extracted only for the first sequence of crossover trials to address potential bias arising from the carry‐over effects of one period to a subsequent trial period and the possibility of ‘period effects’ for crossover trials (Higgins et al., 2022). Studies from inception to November 2022 in any language were searched. Studies focused on search tools that are no longer available or outdated educational interventions such as Grateful med (Dorsch et al., 2022), electronic textbooks, and CD‐ROMs were excluded.

The populations in this review included post‐registration healthcare professionals, as defined by the Health and Welfare chapter of the ISCED‐F 2013 (2015). Healthcare professionals working in any healthcare institution settings in either private or public sectors were included in this review.

### Outcome measures

The outcomes of the studies were categorised according to Miller's classification of clinical competence to assess participants' knowledge and skills by the type of assessment utilised (Miller, 1990). For instance, if an outcome reported as ‘skill’ was evaluated by a knowledge test, it was considered that the outcome was knowledge, independently of the reported outcome. Participants' attitudes and levels of satisfaction were analysed independently.

#### Primary outcomes

The following primary outcomes were evaluated:

1. Participants' ‘post‐intervention knowledge’ is assumed to objectively evaluate participants' conceptual understanding. It was assessed whether the tools were validated or non‐validated. If multiple post‐test assessments were conducted, the first post‐test assessment in the analysis was used.
2. The EBM model consists of five steps: (1) formulating answerable clinical questions; (2) finding the evidence; (3) appraising the evidence; (4) applying the evidence; and (5) evaluating performance (Sackett, 1997). In this review, participants' ‘post‐intervention skills’ are defined as the participants' ability to execute the first two steps of the EBM model: (1) formulating answerable clinical questions and (2) finding the evidence. It was assessed whether the tools were validated or non‐validated.
  - ‘Search duration’ is the time for conducting a single search (Ho et al., 2016).
  - ‘Search recall’ is the participants' ability to retrieve articles relevant to the research question (DeMars & Perruso, 2022; Lowe & Barnett, 1994; Riesenberg & Justice, 2014).
  - ‘Search precision’ is the participants' ability to eliminate irrelevant articles (DeMars & Perruso, 2022; Lowe & Barnett, 1994; Riesenberg & Justice, 2014).
  - ‘Number of successful searches’ is defined where the referenced study utilised valid methodology, where the population studied applied to the question, and where the abstract included quantitative data to answer the specific question (Ho et al., 2016).
  - ‘Number of questions’ answered is defined as the total number of questions for which participants found an answer.
3. Participants' ‘post‐intervention attitude’ is the participants' perceptions about acquiring new knowledge and skills in relation to the intervention or patient care. It was assessed whether the tools were validated or non‐validated.
4. Participants' ‘post‐intervention satisfaction’ is defined as participants' levels of expectation towards the intervention. It was assessed whether the tools were validated or non‐validated.

#### Secondary outcomes

The following secondary outcomes were also evaluated:

1. Participants' ‘post‐intervention behaviour change’ is any change in how participants modify their practice. It was assessed whether the tools were validated or non‐validated.
2. Patient‐related outcomes include quality of life, morbidity, mortality rates, and more. They were assessed whether the tools were validated or non‐validated.
3. Cost and cost‐effectiveness of implementing the educational interventions.
4. Adverse effects of the educational intervention, such as inappropriate patient treatment.

### Identification of studies

The search strategy included MEDLINE (Ovid), Cochrane Central Register of Controlled Trials (CENTRAL; Cochrane Library), EMBASE (Ovid), Web of Science, Cumulative Index of Nursing and Allied Health Literature (CINAHL; EBSCO), and the first 10 pages of the Google Scholar search engine. All databases were searched for studies irrespective of publication year and without language restriction. The structure adopted was based on a PICO‐style approach. A medical librarian developed it through a discussion with a study team member (L.M.M.). Supporting Information S1 presents the MEDLINE search strategy. The search strategy was developed for MEDLINE and then adapted for other databases. The WHO International Clinical Trials Registry Platform (ICTRP) portal and ClinicalTrials.gov were searched for unpublished clinical trials to mitigate publication bias. Finally, the reference lists of all included studies and relevant systematic reviews were examined. The authors were contacted to request clarification if data retrieved from the published studies were incomplete or missing. The search results from all databases were imported into a single EndNote 20 (Clarivate) library, and duplicate records were removed.

**FIGURE 1:**
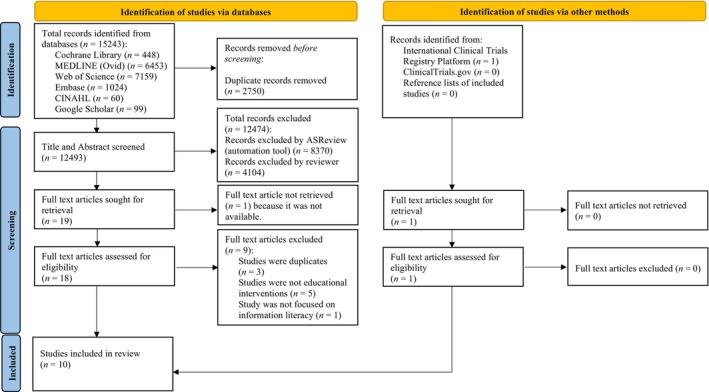
Preferred Reporting Items for Systematic Reviews and Meta‐Analyses flow diagram. [Colour figure can be viewed at wileyonlinelibrary.com]

The first reviewer (L.M.M.) independently screened titles and abstracts on ASReview (ASReview LAB developers, 2022) to identify studies that potentially meet the inclusion criteria. Only 33% of the titles and abstracts were screened, following a rule that was pre‐determined and adhered to before screening commenced on ASReview (ASReview LAB developers, 2022). This rule was set based on a study that found that 95% of eligible studies are found after screening between 8% and 33% of studies on ASReview (van de Schoot et al., 2021). Two reviewers (L.M.M. and L.X.W.) working in parallel independently retrieved and assessed the full‐text versions of selected articles. Reviewers' results at each step of the screening process were compared; disagreements were settled between them or with a senior researcher (L.T.C.) if an agreement could not be reached. The screening process steps were presented in a flow diagram according to PRISMA guidelines, including the reasons for exclusion at the full‐text screening stage (Figure 1).

### Data extraction and quality assessment

Working independently, according to Cochrane methodology, two reviewers (L.M.M. and L.X.W.) extracted the data for all included studies using a pre‐piloted Microsoft Excel data recording form (Higgins et al., 2022). The information extracted included the following: study design and participants' demographics, type of educational intervention, the method used to deliver the intervention, that is, precisely what was delivered, who delivered it, how it was delivered, where it was delivered, how much was delivered, and whether the intervention was generalisable (Hoffmann et al., 2014). Characteristics of interventions were grouped into delivery platforms, such as online, face‐to‐face, and both, and delivery format (lecture, workshops, small groups, computer‐assisted, self‐directed, online learning, or bedside education). Disagreements between the reviewers were resolved through consensus or consultation with a third reviewer, a senior researcher (L.T.C.).

The two reviewers assessed the methodological quality of included studies in parallel with Cochrane's Risk of Bias tool (Higgins et al., 2022). The following domains were assessed: the randomisation process; deviations from intended interventions; missing outcome data; measurement of the outcome; and selection of the reported result. The blinding of participants or personnel was not assessed, as the nature of the intervention precludes blinding. The results were reported using a risk‐of‐bias table per the Cochrane Handbook for Systematic Reviews of Interventions (Higgins et al., 2022).

### Statistical analysis

RevMan 5.4.1 software was used for preparing and maintaining Cochrane Reviews to analyse the data. To estimate the effect size of the educational interventions in the primary study, the mean difference and 95% CI were calculated if the results were reported as a continuous variable or the risk ratio and 95% CI were calculated when the study reported the outcome as a dichotomous variable. If the same outcome was reported by more than one study using different types of variables, the mean differences were recalculated into standardised mean differences.

When relevant outcome data were missing from a primary study, an attempt to obtain the information by contacting the study authors was made. If a response was not received in 2 weeks, it was reported accordingly. Whenever possible, analyses were conducted on an intention‐to‐treat basis.

### Data synthesis

The articles were grouped according to study design, outcome, and type of comparison. Studies that did not have sufficient statistical parameters were analysed in a narrative synthesis.

Before attempting the meta‐analysis, the statistical heterogeneity was assessed by calculating the *I* ^2^ statistic. A random‐effects model was employed for our meta‐analysis, which assumes that the various studies estimate different yet related intervention effects. This approach could explain heterogeneity that cannot be explained otherwise. Due to limited data, a sensitivity or subgroup analysis was not conducted.

## RESULTS

### Results of the search

The search strategy focusing on educational interventions for improving information literacy in healthcare professionals identified 15,243 unique references. Of these, 2750 duplicate references were removed. Upon screening of titles and abstracts, 12,474 ineligible references were excluded, and 19 potentially eligible full‐text studies were retrieved. The ASReview excluded 8370 records and the other 4104 records were excluded by the first reviewer after 4123 (33%) of the titles and abstracts were screened. Out of 19 potentially eligible full‐text studies identified via databases, one could not be retrieved. Of the 18 full‐text studies assessed for eligibility identified via databases, nine studies that did not meet the inclusion criteria were excluded: five were not comparing educational interventions, three were duplicates, and one was not focused on information literacy (Supporting Information S2). One study was identified from a registry platform, assessed for eligibility, and included in this review. Therefore, a total of 10 studies (1458 healthcare professionals) were included in the review; one randomised crossover trial and nine RCTs (Bradley et al., 2002; Cabell et al., 2001; Cheng, 2003; Eldredge et al., 2008; Erickson & Warner, 1998; Haynes et al., 1993; Hoogendam et al., 2012; Pearce‐Smith, 2006; Stark et al., 2007; Villanueva et al., 2001). The flow of studies through the systematic review process is shown in Figure 1. Characteristics of the 10 included studies are summarised in Table 1.

**TABLE 1:** Characteristics of included studies.

Study, country (design)	Participants (*N*) and details	Study aims	Content description	Intervention	Control	Learning outcomes
Bradley et al. (2002), United States (RCT)	10 Neonatal intensive care unit residents on 1‐month rotation	To evaluate whether real‐time instruction and feedback by medical librarians improved EBM searching in OVID MEDLINE	Librarians provide feedback for searching in MEDLINE	Lecture Bedside education	Lecture	Skills (recall and precision), attitudes, and satisfaction
Cabell et al. (2001), United States (RCT)	48 Internal medicine residents at Duke University Hospital	To measure an educational intervention's effect on the EBM process's first steps, moving from a clinical question to a medical literature search	One‐hour didactic sessions conducted by the principal investigator and medical librarian, using well‐built clinical question cards and practical experience supervised by the chief resident on building questions, were provided	Lecture Bedside education Small‐group discussion	Lecture	Skills (Number of log‐on to Medline, searching volume, abstracts, and full‐text viewed, and time spent searching)
Cheng (2003), Hong Kong (RCT)	800 Doctors, nurses, and allied health professionals	To test if a 3‐h educational workshop is more effective (than no training) in improving clinical question formulation, information‐seeking skills, knowledge, attitudes, and search outcomes	Supervised hands‐on practice and feedback from librarians were provided	Lecture Bedside education Computer‐assisted learning	Self‐directed^a^	Knowledge, attitudes, and satisfaction
Eldredge et al. (2008), United States (RCT)	93 Administrators, disease prevention specialists, epidemiologists, health educators, nurses, nutritionists, physicians, program directors, and social workers from the New Mexico Department of Health	To determine whether a 3‐h library and informatics training (did not provide details on the trainer), emphasising PubMed searching skills, increased the frequency and sophistication of participants' practice‐related questions	The training was focused on searching in PubMed	Lecture	No training	Search skills, increased frequency, and sophistication of practice‐related questions
Erickson and Warner (1998), United States (RCT)	31 Obstetrics and gynaecology residents at an academic medical centre	To ascertain a librarian's individual 1‐h tutorial session's impact on MEDLINE utilisation among obstetrics and gynaecology residents	The supervised hands‐on practice was provided	Lecture Bedside education	No training	Skills (recall and precision)
Haynes et al. (1993), Canada (RCT)	308 All clinicians and clinicians‐in‐training in Medicine, Paediatrics, Family Medicine, Surgery, Psychiatry, and Obstetrics and Gynaecology departments	To determine if a preceptor and individualised feedback improve physicians' performance by searching MEDLINE in clinical settings	One‐hour lecture and 1‐ h with librarians and preceptors providing feedback for the MEDLINE search	Lecture Bedside education Self‐directed^a^	Lecture Self‐directed^a^	Attitudes, satisfaction, and behaviour change
Hoogendam et al. (2012), The Netherlands, (randomised crossover trial)	22 Specialists from the vascular medicine staff and internal medicine residents from the Radboud University Nijmegen Medical Centre	To determine whether the PICO format was helpful for quick searches of PubMed	An expert searcher explained a 1h lecture on the use of PICO	Extended lecture (with PICO)	Lecture (without PICO)	Skills (recall and precision)
Pearce‐Smith (2006), United Kingdom (RCT)	17 Doctor, nurse, allied health, and manager working within the Oxfordshire Radcliffe Hospitals NHS Trust	To establish whether there is a significant difference in knowledge and skills, between self‐directed learning using a web‐based resource directed by participants, compared with a 2‐h classroom‐based interactive workshop led by a librarian, for teaching health professionals how to search	Supervised hands‐on practice and feedback from librarians were provided	Lecture Bedside education Self‐directed^a^	Self‐directed^a^ online learning	Knowledge
Stark et al. (2007), United States (RCT)	77 Second‐ and third‐year internal medicine residents	To design and implement a database searching tutorial (did not report the duration of the tutorial) for residents on inpatient rotations and to evaluate its impact on residents' skills and comfort by searching MEDLINE and filtered EBM resources	The use of PICO, supervised hands‐on practice and feedback from medical librarians and faculty members were provided	Lecture Small‐group discussion	Lecture	Skills (search duration and number of successful searches)
Villanueva et al. (2001), Australia (RCT)	52 Doctors, nurses, allied health, hospital administration, psychology, and in‐service education units	To determine whether adding simple instructions and examples on clinical question formulation (provided by staff working in the Evidence Centre) would increase the specificity of the question being submitted by the healthcare professional compared with using a standard form without instructions and examples	A brief explanation of the importance of proper question formulation, some written instructions, and a diagrammatic illustration of how dimensional elements may be arranged was provided in the revised form (this revised form was implemented for 2 months)	Self‐directed^a^ (With specific instructions)	Self‐directed^a^	Skills (change in the proportion of reformulated questions that had each of the four dimensions of questions specificity explicitly described)

Abbreviations: PICO, Population, Intervention, Comparison(s), and Outcome; RCT, randomised controlled trial.

^a^ Self‐directed learning indicates participants learning about information literacy independently without any additional support or guidance.

### Characteristics of included studies

All included studies were conducted in high‐income countries, five in the United States (Bradley et al., 2002; Cabell et al., 2001; Eldredge et al., 2008; Erickson & Warner, 1998; Stark et al., 2007), and one each in Australia (Villanueva et al., 2001), Canada (Haynes et al., 1993), Hong Kong (Cheng, 2003), The Netherlands (Hoogendam et al., 2012), and United Kingdom (Pearce‐Smith, 2006). Six studies focused on doctors (Bradley et al., 2002; Cabell et al., 2001; Erickson & Warner, 1998; Haynes et al., 1993; Hoogendam et al., 2012; Stark et al., 2007), and four studies included mixed populations of doctors, nurses, and other healthcare professionals (Cheng, 2003; Eldredge et al., 2008; Pearce‐Smith, 2006; Villanueva et al., 2001). A range of educational interventions was evaluated, including active instruction (by librarians, expert searchers, authors and faculties, tutorials, and lectures). Control group interventions ranged from no formal training to basic lectures on search skills and/or formulation of questions without feedback, attendance at a medical conference, personal searching, use of original search form, limited individual tutorial with the librarians, and the independent use of online resources (see Table 1).

The presentation of outcome data was rarely complete. There was no missing outcome data entered. For instance, studies either had missing or unclear relevant outcome data (Bradley et al., 2002; Villanueva et al., 2001), and due to a lack of response from the authors or their lack of access to the study data, no additional data was collected. Therefore, all the effects of the interventions, except for attitude, could not be estimated by recalculating the data from those studies. Also, there was no information about validation in any of the assessment instruments.

### Risk of bias

**FIGURE 2:**
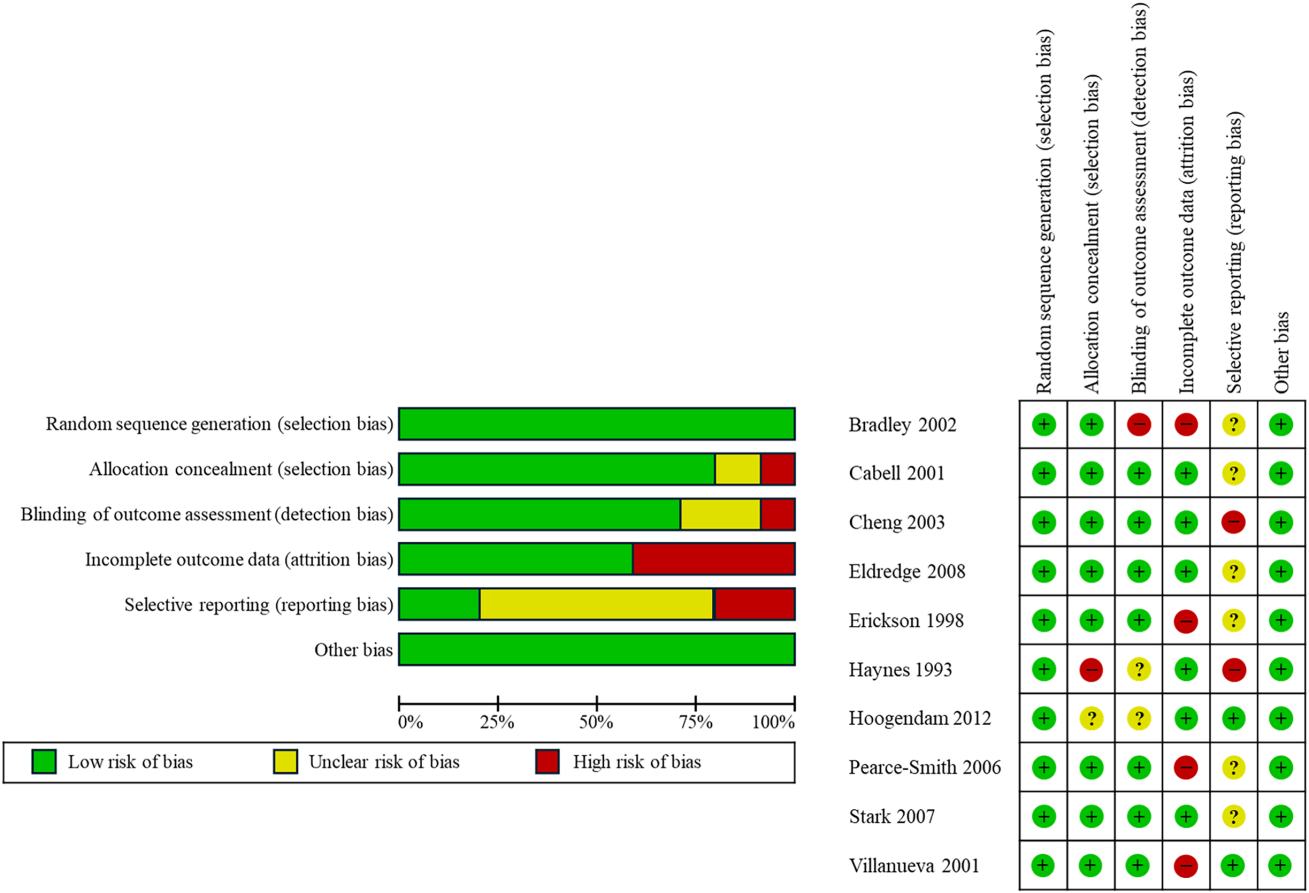
Risk of bias graph and summary. [Colour figure can be viewed at wileyonlinelibrary.com]

Overall, studies were judged to be at a low risk of bias for randomisation. Six studies were regarded as high risk of bias for at least one domain, including allocation concealment, blinding of outcome assessment, incomplete outcome data, and selective reporting (see Figure 2). Eight studies provided sufficient information on allocation concealment. Seven studies were judged to be at low risk of detection bias as the intended blinding was deemed adequate. Seven studies were at low risk of bias for blinding of outcome assessment as they provided detailed information on blinding of outcome measures and/or used predetermined assessment tools (multiple choice questions [MCQs], surveys, etc.). Six studies were at low risk of bias, with four being judged as high risk of bias for attrition bias as they had presented incomplete outcome data (Bradley et al., 2002; Erickson & Warner, 1998; Pearce‐Smith, 2006; Villanueva et al., 2001). Eight studies were considered to be at unclear or high risk of bias for reporting bias as selective outcome reporting was observed (Bradley et al., 2002; Cabell et al., 2001; Cheng, 2003; Eldredge et al., 2008; Erickson & Warner, 1998; Haynes et al., 1993; Pearce‐Smith, 2006; Stark et al., 2007). All studies were also at low risk of bias for ‘other biases’ as no other bias‐related concerns were observed in the included studies.

### Primary outcomes (post‐intervention)

#### Knowledge

A total of two studies (817 participants) (Cheng, 2003; Pearce‐Smith, 2006) assessed post‐intervention knowledge using MCQs (Cheng, 2003; Pearce‐Smith, 2006) or true‐false questions (Cheng, 2003). One study reported knowledge outcomes (i.e., understanding of Boolean logic, how to improve specificity and sensitivity for clinically sound studies) and compared the use of lectures, bedside education, and computer‐assisted learning with a control group of self‐directed learning (Cheng, 2003). The study only reported the findings narratively using the *p‐*value indicating that the intervention group scored significantly better than the control group with *p* = 0.000 (Cheng, 2003). Another study (17 participants) compared lecture, bedside education, and self‐directed learning with a control group of online learning. It evaluated knowledge using MCQs to determine the post‐intervention question formulation, search strategy, and citation selection scores. The study reported no significant difference between the intervention and control groups on the number of correct MCQ answers (MD = 1.37; 95% CI −0.77 to 3.51) (Pearce‐Smith, 2006).

#### Skill

Four studies (140 participants) assessed post‐intervention skills using search duration (Stark et al., 2007), search recall (Bradley et al., 2002; Erickson & Warner, 1998; Hoogendam et al., 2012), search precision (Bradley et al., 2002; Erickson & Warner, 1998; Hoogendam et al., 2012), and the number of successful searches (Stark et al., 2007). Only one study (77 participants) compared lecture and small‐group discussions with a control group of lectures only. It reported no difference between the intervention and control group in the search duration (MD = −0.84; 95% CI −1.96 to 0.28) and the number of successful searches (MD = 0.39; 95% CI −0.30 to 1.08) (Stark et al., 2007).

Three studies (63 participants) assessed skills using search recall and search precision (Bradley et al., 2002; Erickson & Warner, 1998; Hoogendam et al., 2012). One study compared a lecture and bedside education with a control group of lectures only (Bradley et al., 2002). However, the study only presented the mean search recall in both arms reporting that the intervention group did not retrieve any of the 13 articles deemed relevant to the research question, compared with control group members who retrieved 2 of the 13 articles (Bradley et al., 2002). In addition, the study did not report the post‐intervention search precision result for the intervention group (Bradley et al., 2002). Another study compared lecture and bedside education with a control group with no training (Erickson & Warner, 1998). However, the study only presented the mean search recall and precision as 25% and 40%, respectively, with no information on how many articles this was based on (Erickson & Warner, 1998). Finally, one study (22 participants) compared the use of extended lectures with a control group of basic lecture and evaluated skill using the search recall (MD = 1.35; 95% CI −39.26 to 41.96) and search precision (MD = −0.58; 95% CI −2.39 to 1.23) (Hoogendam et al., 2012). The study reported no significant difference in the mean search recall and precision between the intervention group using the PICO format and the control group using the non‐PICO format for quick searches on PubMed (Hoogendam et al., 2012).

#### Attitude towards the intervention

**FIGURE 3:**
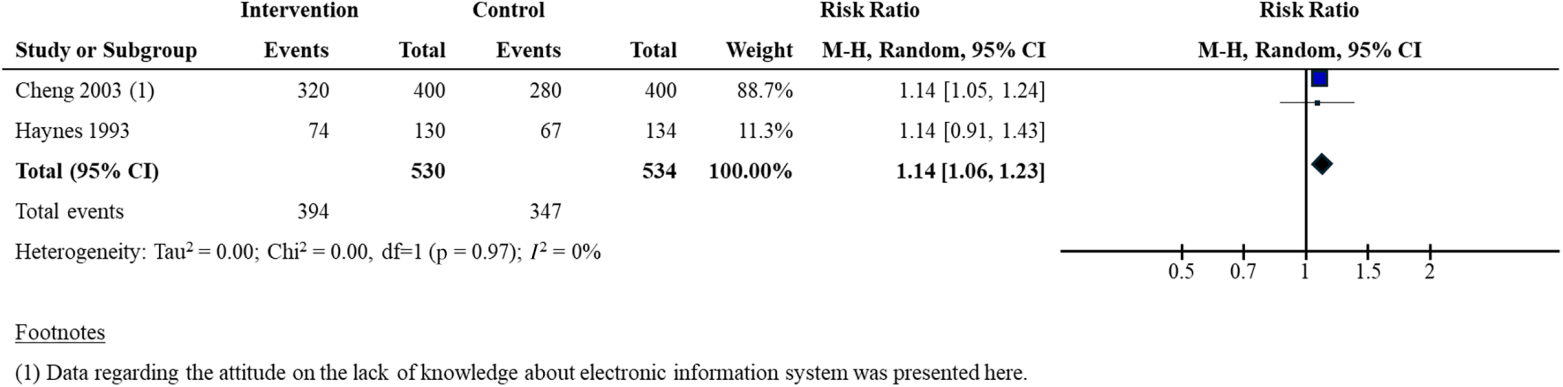
Forest plot for the attitude outcome (post‐intervention). df, degrees of freedom; M‐H, Mantel–Haenszel method; Random, Random effects model. [Colour figure can be viewed at wileyonlinelibrary.com]

Three studies (1118 participants) (Bradley et al., 2002;Cheng, 2003; Haynes et al., 1993) assessed attitude using questionnaires (Bradley et al., 2002; Cheng, 2003; Haynes et al., 1993). One study reported attitude outcomes using questions (i.e., ability to find relevant patient care information, confidence in finding adequate search terms in MEDLINE, and ability to formulate effective search strategies in MEDLINE) and compared the use of lecture and bedside education with a control group of lectures only (Bradley et al., 2002). However, the study only narratively presented the average score of the questions derived using a five‐point scale (with one indicating strong disagreement and five indicating strong agreement) (Bradley et al., 2002). The study reported that the attitudes on their abilities to formulate effective search strategies in MEDLINE had an average score of 4.2 for the intervention group while the control group had an average score of 2.8 (Bradley et al., 2002). This indicated that the control group found developing search strategies more difficult after the intervention. Two studies with dichotomous data comparing interventions (including lecture, bedside education, and computer‐assisted/self‐directed learning) with the control group (mainly self‐directed learning) (Cheng, 2003; Haynes et al., 1993) favoured the control groups' intervention (RR: 1.14; 95% CI 1.06–1.23; *N* = 2 studies; 1064 participants; *I* ^2^ = 0%; moderate certainty evidence; see Figure 3).

#### Satisfaction

Three studies (1118 participants) assessed satisfaction using questionnaires (Bradley et al., 2002; Haynes et al., 1993) and scaled ratings (Cheng, 2003). One study compared the impact of lecture and bedside education with a control group of lectures only using a five‐point scale (with one indicating strong disagreement and five indicating strong agreement) to determine participants' satisfaction with their searching skills. The study only presented the average score for the question on satisfaction with searching skills, where the intervention group had a mean score of 2.0, and the control group had a mean score of 2.6 (Bradley et al., 2002). Another study compared lectures, bedside education, and computer‐assisted learning with control of self‐directed learning and measured satisfaction using a rating scale to determine participants' immediate subjective assessment of the workshop (Cheng, 2003). The study only reported that 96% of the intervention group were satisfied with the intervention, as the question on satisfaction rating for the intervention was omitted for the control group, who only did self‐directed learning (Cheng, 2003). Finally, one study (308 participants) with dichotomous data reported that it is about 40% less likely to find the additional bedside education (specifically librarian feedback) in the intervention group helpful compared with a control group of self‐directed learning (RR: 0.84; 95% CI 0.70–1.01) (Haynes et al., 1993).

### Secondary outcomes

Only one study (308 participants) reported on the self‐reported behaviour change outcome using a questionnaire to determine how searches done for patient‐care questions affected a clinical decision (Haynes et al., 1993). The study compared lecture, bedside education, and self‐directed learning with a control group of lecture and self‐directed learning (Haynes et al., 1993). They reported that 44% of searches done for patient‐care questions affected a clinical decision in the intervention group compared with 35% in the control group. But the difference between the groups was not statistically significant (MD = 9%; 95% CI −3 to 19) (Haynes et al., 1993). No studies of other secondary outcomes (including patient‐related outcomes, cost and cost‐effectiveness of implementing the education interventions, and adverse effects of the educational intervention) were reported.

## DISCUSSION

### Overview

Our systematic review included 10 studies that evaluated the effectiveness of educational interventions for improving information literacy exclusively in healthcare professionals. This review found that the existing evidence on the impact of these educational interventions on healthcare professionals' knowledge, skill, and satisfaction was inconclusive primarily because of incomplete outcome data. The included studies did report an improvement in attitudes among healthcare professionals taking part in lectures compared with having additional bedside education or computer‐assisted learning. Studies should include a wider set of outcomes, be reported better and explore the use of digital technology for delivery of educational interventions. Our findings should be interpreted with caution given the small number of studies included in this review, their limitations of reported outcomes with a high or unclear risk of bias and focused on the search of PubMed and Medline.

Studies on information literacy training in healthcare professionals have become less common after 2012, possibly due to a shift towards integrating information literacy training into medical education. As such, information literacy is one of the required competencies of healthcare professionals as part of their undergraduate and postgraduate medical education (Collins et al., 2007; Frank, 2005; Sezer, 2020; Simons et al., 2012; Swing, 2007). Nevertheless, health professionals must still use evidence in everyday clinical practice (Heselmans et al., 2009; Lafuente‐Lafuente et al., 2019). However, several barriers prevent healthcare professionals from seeking the best scientific evidence (Brassil et al., 2017; Ely et al., 2002; Green & Ruff, 2005; Sadeghi‐Bazargani et al., 2014). They include a lack of confidence in developing good clinical questions, an appropriate search strategy to identify relevant medical literature, a lack of experience using literature databases, and information overload (Brassil et al., 2017; Ely et al., 2002; Green & Ruff, 2005; Lafuente‐Lafuente et al., 2019; Sadeghi‐Bazargani et al., 2014; Sbaffi et al., 2020). Many healthcare professionals have only rudimentary formal training in conducting literature searches and critically evaluating studies. Medical and healthcare education programmes may emphasise clinical skills and patient care, with a greater emphasis on clinical skills and patient care, with less emphasis placed on how to conduct literature research (Brassil et al., 2017; Ely et al., 2002; Green & Ruff, 2005; Lafuente‐Lafuente et al., 2019; Sadeghi‐Bazargani et al., 2014; Sbaffi et al., 2020). As a result, information literacy training should complement the proliferation of information sources and address the reported barriers. Future research should be conducted to determine whether healthcare professionals in the clinical setting could benefit from information literacy refresher training.

Another potential reason behind the limited and dated evidence on information literacy education of healthcare professionals could be a shift in the learning needs of healthcare professionals for improving information literacy. For example, in line with the proliferation of the usage of smartphones among healthcare professionals (Boulos et al., 2011), coupled with the availability of evidence‐based information summaries on mobile apps (Johnson et al., 2016), healthcare professionals' learning needs and aims for improving information literacy may have changed. The information literacy training of healthcare professionals may need to focus on the ability to formulate the question and search for appropriate pre‐appraised evidence rather than primary studies in electronic databases such as PubMed. Evidence‐based information summaries are pre‐appraised and regularly updated by experts to ensure the most recent evidence is included (Brian Haynes, 2006). Busy clinicians can save time with condensed research evidence summaries. Studies have shown that healthcare professionals increasingly use such medical apps clinically at the point‐of‐care (Al‐Ghamdi, 2018; Hedhli et al., 2021; Liu et al., 2016; Ozdalga et al., 2012). Nonetheless, the quality and accuracy of evidence‐based information summaries can vary (Mauricette et al., 2023). The information literacy training of healthcare professionals, including medical trainees, may need to focus on the ability to formulate the question and search for appropriate pre‐appraised evidence rather than primary studies in electronic databases such as PubMed. In this case, the educational intervention's aim remains focused on the first two steps of the EBM model. As a result of the emergence of pre‐appraised evidence, health libraries, and information professionals should incorporate pre‐appraised evidence searching and use training for clinicians in their information literacy training. It would be beneficial if librarians assessed the information literacy training they provide to healthcare professionals. It is still unknown what works to improve information literacy, and we still do not know how to measure reliable information literacy training outcomes. However, the type of educational intervention may differ and should focus on greater use of smartphones for both the delivery of training and access to evidence. Although smartphone apps such as UpToDate and DynaMed are regarded as accessible, user‐friendly, evidence‐based sources of information, healthcare professionals might still benefit from training on how and when to use these tools in their clinical practice as well as on how to run additional searches of the literature (Johnson et al., 2016). Clinicians must be well‐versed in information literacy, particularly when searching for evidence on rare or new medical conditions that may not have been curated for evidence synthesis.

None of the studies in our review used validated outcome measures to evaluate the effectiveness of educational interventions for improving information literacy. This aligns with a systematic review's findings that reported that out of 104 unique EBM assessment tools, most of the tools had not been validated (Shaneyfelt et al., 2006). The Fresno test and Berlin Questionnaire were the only validated tools that evaluated all EBM steps (Shaneyfelt et al., 2006). Therefore, future research on information literacy in healthcare professionals should also encompass the development of validated assessment tools specific to the learning needs of the respective healthcare professionals.

### Strengths and weaknesses

To the best of our knowledge, this is the first systematic review to evaluate the effectiveness of educational interventions for information literacy exclusively in healthcare professionals. A comprehensive search across different databases with no language limitations, including grey literature sources, followed the Cochrane gold standard methodology, which attempted to minimise the risk of bias and errors in the review process. Additional steps to identify unpublished studies, such as searching trial registers, and PhD theses, screening references of included studies, and contacting one abstract author for further information, were included. It was not possible to formally assess the risk of publication bias because of the small number of heterogeneous studies included in our review. Still, given our extensive search, it is unlikely that relevant studies have been missed up until November 2022. Moreover, two independent reviewers were involved in all stages of the review process, standardised data extraction forms were used, and an accepted tool to assess the risk of bias in the included studies was employed. Overall, the risk of bias for most studies was considered low, with some instances of potentially high risk of attrition and reporting bias identified. The quality of evidence is moderate for attitude because of the unclear and high risks of bias and inconsistency, that is, heterogeneity in the study results and types of participants, interventions, and outcome measurement instruments (Higgins et al., 2022).

Our review also has several limitations. The small number of included studies meant that it was not possible to carry out any subgroup analyses or assess the risk of publication bias. Therefore, the likelihood of publication bias cannot be ruled out in this case. Another possibility is that, in this study's context, RCTs and crossover trials are uncommon. Hence, the limited included studies. However, there might be other types of research available in this area. This review could have been constrained by the incomplete data and the absence of studies listed under other terms. The studies used non‐validated measurement instruments to measure outcomes, making comparing educational interventions between settings challenging (Squires et al., 2019). Next, understanding the basic principles and functionalities of the ASReview tool is required for its use. However, with the right resources and community support, this barrier can be surmounted. Finally, we did not include studies or reviews on educational interventions that spanned several EBM steps and could have provided insights into steps one and two. However, such reviews, though recently published, did not include studies that were focused on only the first two steps of EBM. Also, considering that the outcome measures of such studies would focus on general knowledge, abilities, and attitudes with all five steps, this may not have influenced the results of our study. Nevertheless, the systematic review conducted by (Portela Dos Santos et al., 2022) offers additional, more recent evidence pertinent to this field. This systematic review suggests that effective educational strategies for evidence‐based practice (EBP) are crucial, with computer‐based learning being the most cost‐effective and efficient approach. Therefore, while developing an effective educational strategy for EBP appears promising, it is important to note that the studies reviewed do not adequately address the initial two steps.

### Implications for practice and research

Further research should conduct well‐designed RCTs to evaluate outcomes such as knowledge, skills, attitude, satisfaction, patient‐related outcomes, cost‐effectiveness, adverse effects of the educational intervention, and behaviour change. There is a need to standardise the methods for reporting meaningful and specific data. Future studies could be designed with larger, appropriately powered RCTs in low‐ and middle‐income countries to ensure better representation. It is pertinent to the development of validated outcome‐assessment tools. The fact that clinicians have access to an increasing amount of pre‐appraisal evidence, means that future studies should consider the educational intervention focusing on the EBM model's first two steps of searching for primary studies and information summaries. Health libraries and information professionals should incorporate pre‐appraised evidence searching and use training for clinicians in their information literacy training.

There is a need for a multidisciplinary team. Health librarians and information professionals may seek to collaborate with medical education researchers or other researchers and colleagues at their institutions or other institutions on a multicentre study. They could consider including a clinical trials unit in their investigation to ensure methodological robustness. Health libraries and information professionals could begin with a pilot trial before moving to a full RCT. They should measure various outcomes and, if possible, employ validated outcome measurement instruments. Health libraries and information professionals could also track the retention of the participants in their studies. They could include various healthcare professionals in their research and may choose to co‐design the study with participants. Finally, health libraries and information professionals may also consider comparing several types of digital educational interventions.

## CONCLUSIONS

An essential part of EBM is the ability to develop answerable questions based on issues encountered in clinical practice and then to find the best available relevant scientific evidence to answer them. However, there is inconclusive evidence on the effectiveness of educational interventions to help healthcare professionals develop their information literacy skills. There is a need for novel studies in this area to incorporate digital technology in education and for access to information at the point‐of‐care. Future studies should include more comprehensive outcomes, report them in greater detail, and use validated outcome measurement tools.

## CONFLICT OF INTEREST STATEMENT

The authors declare no conflicts of interest.

## Supporting information


**Data S1:** Supporting Information 1.

**Data S2:** Supporting Information 2.

## Data Availability

The data that support the findings of this study are available on request from the corresponding author. The data are not publicly available due to privacy or ethical restrictions.
