# Supplementary material for: Effectiveness of educational interventions for improving healthcare professionals' information literacy: A systematic review
Source: Health Info Libr J. 2025 Feb 2;42(2):131–47. doi: 10.1111/hir.12562 (PMC12723344; doi:10.1111/hir.12562)
Supplement: Supplementary file 1 — Data S1: Supporting Information 1. [file HIR-42-131-s002.doc]

Database: Ovid MEDLINE(R) and Epub Ahead of Print, In-Process, In-Data-Review & Other Non-Indexed Citations, Daily and Versions <1946 to November 22, 2022>

Search Strategy:

--------------------------------------------------------------------------------

1 exp Health Personnel/ (596838)

2 ("health personnel" or "healthcare personnel" or "health care personnel" or "healthcare professional?" or "health care professional?" or "healthcare provider?" or "health care provider?" or "healthcare worker?" or "health care worker?" or doctor? or nurse? or resident? or physician? or clinician?).ab,ti. (1349007)

3 1 or 2 (1691388)

4 Information Literacy/ or "Information Storage and Retrieval"/ or Information Seeking Behavior/ (24713)

5 "information literacy".ab,ti. (481)

6 (search* adj1 (skill? or literature or database? or MEDLINE or Pubmed or evidence* or strateg* or query or queries)).ab,ti. (146666)

7 4 or 5 or 6 (170287)

8 3 and 7 (21701)

9 randomized controlled trial.pt. (581532)

10 controlled clinical trial.pt. (95109)

11 randomized.ab. (583610)

12 placebo.ab. (233593)

13 drug therapy.fs. (2551524)

14 randomly.ab. (396196)

15 trial.ab. (625161)

16 groups.ab. (2439159)

17 9 or 10 or 11 or 12 or 13 or 14 or 15 or 16 (5520439)

18 exp animals/ not humans.sh. (5069381)

19 17 not 18 (4811969)

20 8 and 19 (6453)

***************************
