# Supplementary material for: Effectiveness of educational interventions for improving healthcare professionals' information literacy: A systematic review
Source: Health Info Libr J. 2025 Feb 2;42(2):131–47. doi: 10.1111/hir.12562 (PMC12723344; doi:10.1111/hir.12562)
Supplement: Supplementary file 2 — Data S2: Supporting Information 2. [file HIR-42-131-s001.docx]

**List of excluded full text studies**

Educational intervention not focused on information literacy:

1. Evaluating the impact of an evidence-based medicine educational intervention on primary care doctors' attitudes, knowledge and clinical behaviour: a controlled trial and before and after study <https://doi.org/10.1111/j.1365-2753.2007.00859.x>

Not educational interventions:

1. Utilization of the PICO framework to improve searching PubMed for clinical questions <https://bmcmedinformdecismak.biomedcentral.com/articles/10.1186/1472-6947-7-16>
2. Randomized trial for answers to clinical questions: evaluating a pre-appraised versus a MEDLINE search protocol <https://pubmed.ncbi.nlm.nih.gov/17082828/>
3. Translating Clinical Questions by Physicians Into Searchable Queries: Analytical Survey Study <https://pubmed.ncbi.nlm.nih.gov/32310137/>
4. To Compare PubMed Clinical Queries and UpToDate in teaching Information Mastery to Clinical Residents: A Crossover Randomized Controlled Trial <https://pubmed.ncbi.nlm.nih.gov/21858142/>
5. Effectiveness of bibliographic searches performed by paediatric residents and interns assisted by librarians. A randomised controlled trial <https://pubmed.ncbi.nlm.nih.gov/22051126/>

Duplicates:

1. Resident utilization of information technology <https://www.ncbi.nlm.nih.gov/pmc/articles/PMC1495306/>
2. Educational workshop improved information-seeking skills, knowledge, attitudes and the search outcome of hospital clinicians: a randomised controlled trial <https://pubmed.ncbi.nlm.nih.gov/12757433/>
3. Pediatric Residents and Interns in an Italian Hospital Perform Improved Bibliographic Searches when Assisted by a Biomedical Librarian <https://journals.library.ualberta.ca/eblip/index.php/EBLIP/article/view/18541>
